# C9ORF72 suppresses JAK-STAT mediated inflammation

**DOI:** 10.1016/j.isci.2023.106579

**Published:** 2023-04-06

**Authors:** Weilun Pang, Fenghua Hu

**Affiliations:** 1 Department of Molecular Biology and Genetics, Weill Institute for Cell and Molecular Biology, Cornell University, Ithaca, NY 14853, USA

**Keywords:** Molecular biology, Immunity, Cell biology

## Abstract

Hexanucleotide repeat expansion in the gene *C9ORF72* is a leading cause of amyotrophic lateral sclerosis (ALS) and frontotemporal lobar degeneration (FTLD). C9ORF72 deficiency leads to severe inflammatory phenotypes in mice, but exactly how C9ORF72 regulates inflammation remains to be fully elucidated. Here, we report that loss of C9ORF72 leads to the hyperactivation of the JAK-STAT pathway and an increase in the protein levels of STING, a transmembrane adaptor protein involved in immune signaling in response to cytosolic DNA. Treatment with a JAK inhibitor rescues the enhanced inflammatory phenotypes caused by C9ORF72 deficiency in cell culture and mice. Furthermore, we showed that the ablation of C9ORF72 results in compromised lysosome integrity, which could contribute to the activation of the JAK/STAT-dependent inflammatory responses. In summary, our study identifies a mechanism by which C9ORF72 regulates inflammation, which might facilitate therapeutic development for ALS/FTLD with *C9ORF72* mutations.

## Highlights

• C9ORF72 deficiency leads to hyperactivated TLR3/7/9, cGAS-STING, and JAK-STAT pathways
• Inhibition of JAK1/2 activities rescues inflammation associated with C9ORF72 deficiency
• Lysosome defects caused by C9ORF72 deficiency induces JAK-STAT dependent inflammation

## Graphical abstract

**Figure:**
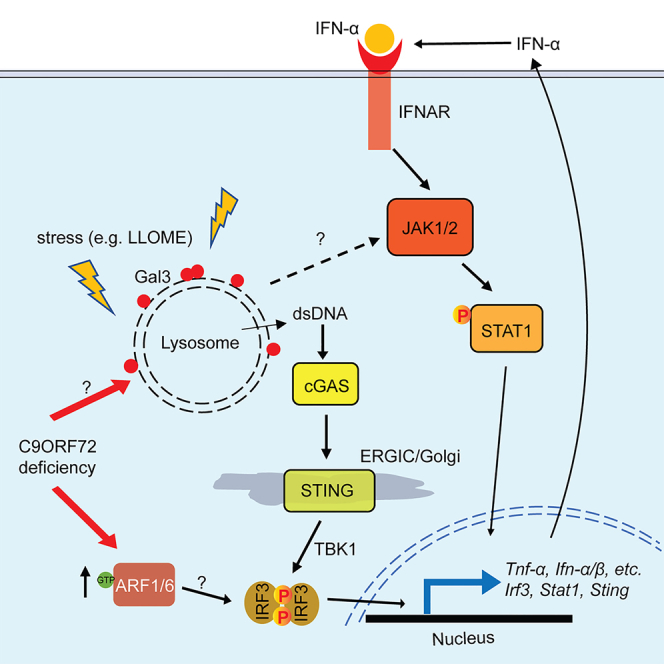

## Introduction

Amyotrophic lateral sclerosis (ALS) and frontotemporal lobar degeneration (FTLD) are two devastating neurodegenerative diseases that belong to the same disease spectrum. These two diseases have overlapping clinical, pathological, and genetic features.^1^^,^^2^ One of the main genetic causes of both ALS and FTLD is hexanucleotide repeat (GGGGCC (G_4_C_2_)) expansion (HRE) in the first intron of the *C9ORF72* gene,^3^^,^^4^ which results in disease phenotypes via both gains of toxicity of RNA repeats and dipeptides and loss of function of the C9ORF72 protein.^5^^,^^6^^,^^7^^,^^8^^,^^9^

While the physiological functions of C9ORF72 remain to be fully elucidated, the most striking phenotype of *C9orf72* knockout mice is enhanced inflammatory responses, resulting in age-dependent lymphadenopathy and splenomegaly.^10^^,^^11^^,^^12^^,^^13^ An increased level of inflammatory cytokines, including TNF-α, IL-1β, IL-6, and IL-10 has been detected in the spleen and serum of *C9orf72* deficient mice.^11^^,^^12^^,^^14^^,^^15^ Patients with *C9ORF72* mutations also have an increased propensity to autoimmune diseases due to the constant, uncontrolled production of inflammatory cytokines.^16^ These studies all suggest that C9ORF72 directly or indirectly regulates one or more inflammatory pathways. Recently, the endosomal toll-like receptor (TLR) and the cGAS-STING signaling pathway have been shown to get upregulated under C9ORF72 deficient conditions.^17^^,^^18^ However, the mechanism that leads to the upregulation of these two innate immune signaling pathways is still unclear.

To explore the mechanism of how the loss of C9ORF72 upregulates inflammatory responses *in vitro* and *in vivo,* we examined inflammatory responses in *C9orf72*^*−/−*^ macrophages and mice. Here, we confirmed that C9ORF72 deficiency leads to an increase in the protein levels of STING and enhanced inflammatory responses. The inhibition of JAK activities rescues the inflammatory phenotypes of C9ORF72 deficient cells and mice. In addition, we demonstrate that lysosome integrity is compromised under C9ORF72 deficient conditions, which leads to JAK-dependent inflammatory activation.

## Results

### Enhanced inflammatory responses under C9ORF72 deficient conditions

**Figure 1:**
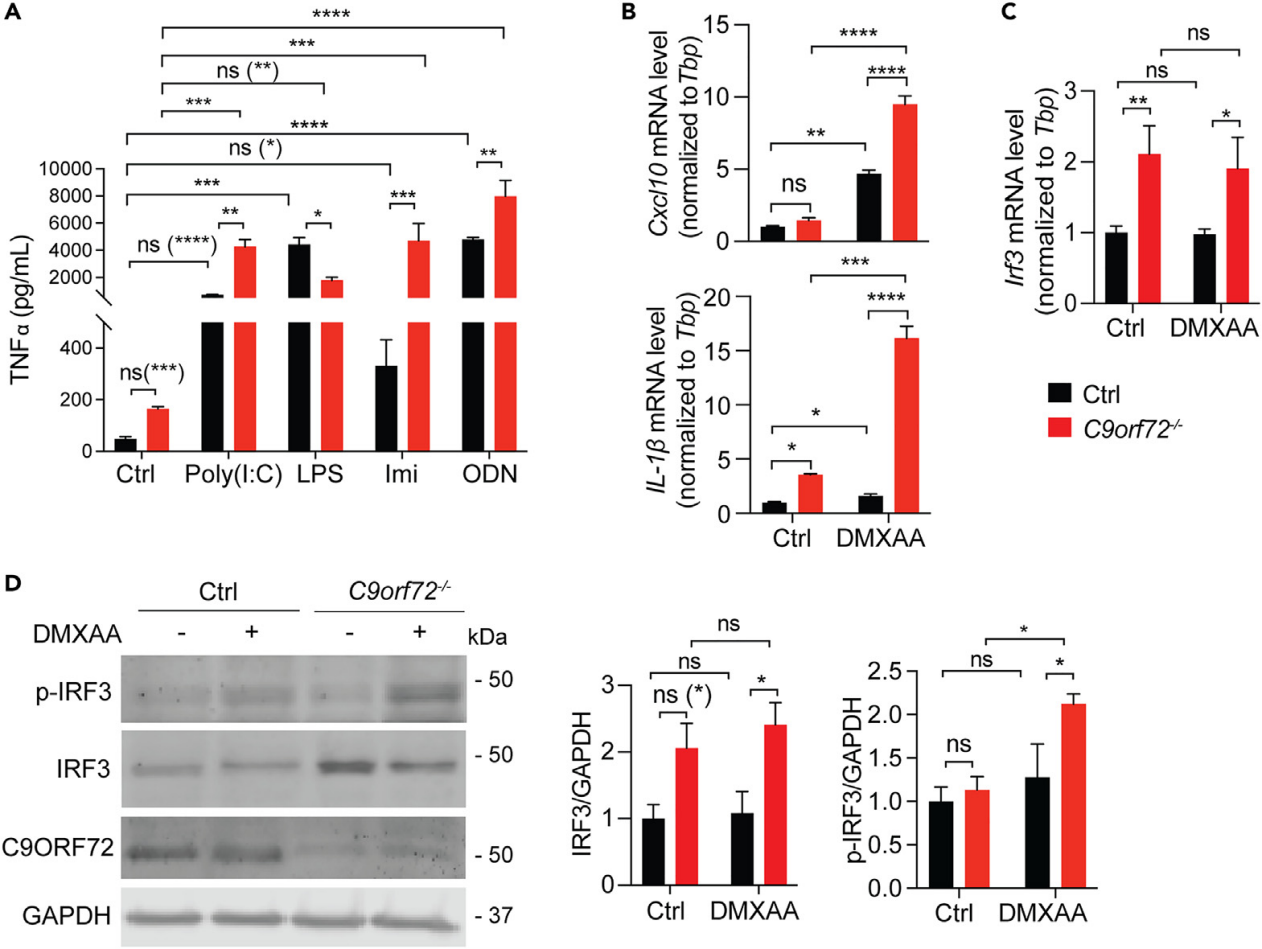
Increased activation of the endosomal TLR and cGAS-STING signaling in *C9orf72*^*−/−*^ cells (A) ELISA analysis of TNFα levels in the cell medium of control and *C9orf72*^*−/−*^ RAW264.7 cells treated 24h with TLR3 ligand poly(I:C) (1 μg/mL), TLR4 ligand LPS (1 μg/mL), TLR7 ligand imiquimod (Imi) (100 μM), or TLR9 ligand CpG ODN (1 μM). Data represent the mean ± SEM. Statistical significance was analyzed by two-way ANOVA (n = 3). Groups that exhibit non-significant differences with two-way ANOVA were re-analyzed using unpaired two-tailed Student’s *t* test, and the results are indicated in parentheses. ns = not significant, ∗p < 0.05, ∗∗p < 0.01, ∗∗∗p < 0.001, ∗∗∗∗p < 0.0001. (B) RT-qPCR measurement of mRNA levels of cytokines *Cxcl10* and *Il1b* in control and *C9orf72*^*−/−*^ RAW264.7 cells without and with 16h of DMXAA (100 μg/mL) treatment. Data represent the mean ± SEM. Statistical significance was analyzed by two-way ANOVA (n = 3), ns = not significant, ∗p < 0.05, ∗∗∗∗p < 0.0001. (C) RT-qPCR measurement of mRNA level of *Irf3* in control and *C9orf72*^*−/−*^ RAW264.7 cells before and after DMXAA treatment. Data represent the mean ± SEM. Statistical significance was analyzed by two-way ANOVA (n = 3). ns = not significant, ∗p < 0.05, ∗∗p < 0.01. (D) Western blot analysis of IRF3 and *p*-IRF3 levels in control and *C9orf72*^*−/−*^ RAW264.7 cells before and after DMXAA treatment. Data represent the mean ± SEM. Statistical significance was analyzed by two-way ANOVA (n = 3). Groups that exhibit non-significant differences with two-way ANOVA were re-analyzed using unpaired two-tailed Student’s *t* test, and the results are indicated in parentheses. ns = not significant, ∗p < 0.05.

C9ORF72 ablation results in lymphadenopathy, splenomegaly, and increased levels of proinflammatory cytokines in mice, supporting the critical role of C9ORF72 in inflammatory responses.^10^^,^^11^^,^^12^ To explore the innate immune response pathway(s) that get activated under *C9orf72* deficient conditions, we ablated *C9orf72* in the macrophage cell line, RAW264.7, using the CRISPR/Cas9 technique.^19^ Control and *C9orf72*^*−/−*^ cells were treated with the toll-like receptor (TLR) ligands, poly (I:C), imiquimod, CpG-ODN, or lipopolysaccharide (LPS), which activate TLR3, TLR7, TLR9, and TLR4 receptors, respectively. The activation of these pathways was examined by measuring the levels of secreted cytokine, TNF-alpha, using ELISA. Consistent with a previous report,^18^ we found the upregulation of endosomal TLR3, TLR7, and TLR9 signaling in the *C9orf72*^*−/−*^ cells compared to control cells, whereas the activities of plasma membrane-localized TLR4 did not exhibit much difference between control and *C9orf72*^*−/−*^ cells after stimulation with its ligand, LPS (Figure 1A). Other than the endosomal Toll-like receptor pathways, the cGAS-STING pathway has also been shown to get upregulated under C9ORF72 deficient conditions.^17^ We confirmed the hyperactivation of the cGAS-STING pathway in *C9orf72*^*−/−*^ cells, as shown by an increase in mRNA levels of proinflammatory cytokines *Cxcl10* and *IL-1b* after treatment of the STING ligand DMXAA (Figure 1B). We also observed an increase in the mRNA and protein levels of *Irf3* (Figures 1C and 1D), a transcriptional factor downstream of STING and other cytosolic DNA/RNA sensors,^20^ in *C9orf72*^*−/−*^ cells. The levels of phosphorylated IRF3 (*p*-IRF3) also exhibit a significant increase in *C9orf72*^*−/−*^ cells after DMXAA treatment (Figure 1D), supporting the hyperactivation of the STING pathway under C9ORF72 deficient conditions.

**Figure 2:**
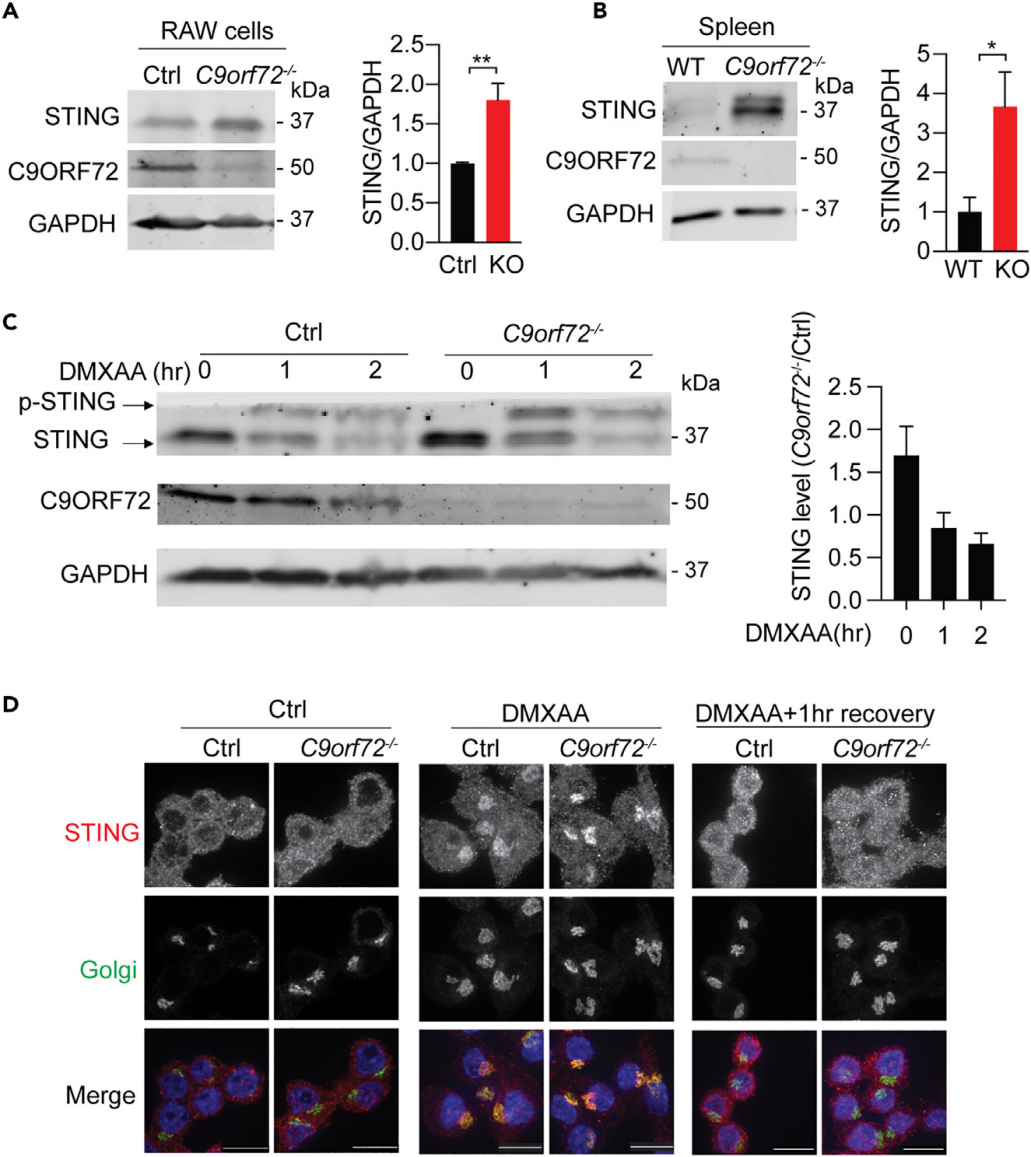
C9ORF72 does not affect STING protein degradation or trafficking (A) Western blot analysis of the protein levels of STING in control and *C9orf72*^*−/−*^ RAW264.7 cells. Data represent the mean ± SEM. Statistical significance was analyzed by unpaired two-tail Student’s *t* test (n = 4), ∗∗p < 0.01. (B) Western blot analysis of the protein levels of STING in spleen lysates from 6-month-old WT and *C9orf72*^*−/−*^ mice. Mixed male and female mice were used. Data represent the mean ± SEM. Statistical significance was analyzed by unpaired two-tail Student’s *t* test (n = 3), ∗p < 0.05. (C) Control and *C9orf72*^*−/−*^ RAW264.7 cells treated with DMXAA for 0, 1, or 2h and STING levels are quantified by western blot. Experiments were repeated three times and a representative western blot was shown. Data represent the mean ± SEM. (D) Control and *C9orf72*^*−/−*^ RAW264.7 cells were untreated, treated with DMXAA for 2h or treated with DMXAA for 2h and allowed to recover for 1 h. Cells were fixed and stained with antibodies against STING and Golgi marker ACBD3 (scale bar = 10 μm). Experiments were repeated three times and representative images were shown.

Since C9ORF72 has been shown to regulate the activities of ARF and RAB GTPases,^21^^,^^22^^,^^23^^,^^24^ key regulators of membrane trafficking, we hypothesized the protein turnover rate of TLRs and STINGs are affected by C9ORF72. Unfortunately, we failed to detect the endogenous TLR proteins in RAW264.7 cells using the commercially available TLR antibodies via western blot or immunostaining. On the other hand, we found that the protein levels of STING are significantly elevated in *C9orf72*^*−/−*^ cells compared to control RAW264.7 cells (Figure 2A). This increase was also observed in the spleen lysates derived from *C9orf72*^*−/−*^ mice (Figure 2B). The increase in STING protein levels under C9ORF72 deficient condition could be due to decreased lysosome degradation, enhanced transcription, and/or trafficking defect(s) of STING. STING is a membrane protein normally localized in the endoplasmic reticulum (ER) and gets translocated to the Golgi apparatus upon activation. Activated STING is then sent to the lysosome for degradation.^25^ To examine whether the degradation of STING has been affected, we treated control and *C9orf72*^*−/−*^ RAW264.7 cells with DMXAA at various time points to induce STING activation and degradation.^26^ Under normal conditions, *C9orf72*^*−/−*^ cells exhibited a 1.6-fold increase in STING levels compared to control cells. The levels of STING decreased significantly after 1 h of DMXAA treatment in both *C9orf72*^*−/−*^ and control cells. After 2 h of treatment, the STING levels were reduced to a similar extent in *C9orf72*^*−/−*^ and control cells (Figure 2C), indicating that STING degradation is not affected by C9ORF72 deficiency. To determine whether C9ORF72 affects STING trafficking, we performed immunostaining to examine the localization of STING in control and *C9orf72*^*−/−*^ RAW264.7 cells. STING shows similar localization in the ER under normal conditions and translocates to the Golgi compartment upon DMXAA treatment in both control and *C9orf72*^*−/−*^ cells (Figure 2D). This Golgi enrichment of STING disappears in both control and *C9orf72*^*−/−*^ cells 1 h after DMXAA removal (Figure 2D). Thus, C9ORF72 deficiency does not have any obvious effect on STING localization and trafficking.

### The JAK-STAT pathway is upregulated under C9ORF72 deficient condition

**Figure 3:**
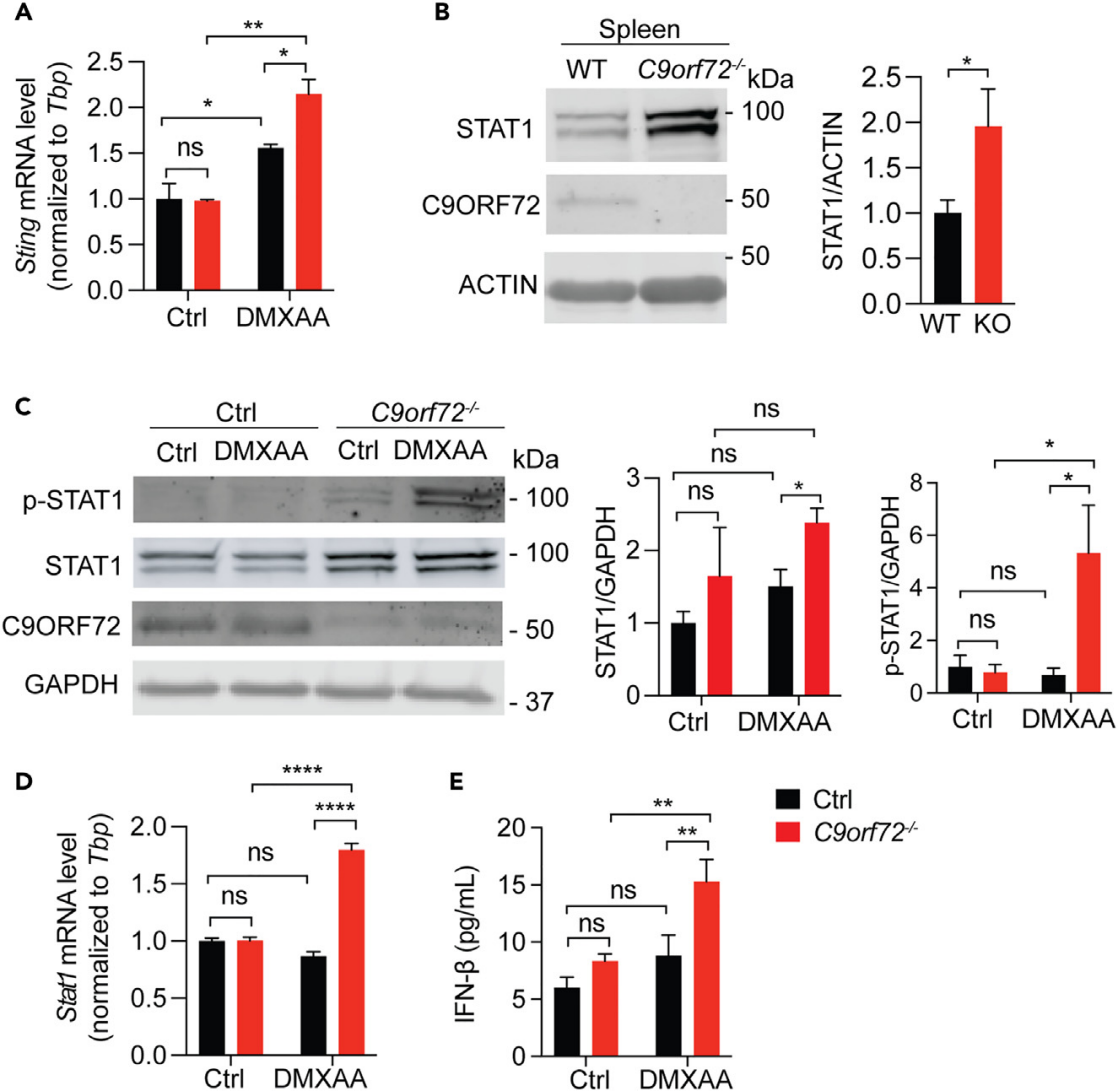
The loss of C9ORF72 leads to JAK-STAT activation (A) RT-qPCR analysis of *Sting* levels in control and *C9orf72*^*−/−*^ RAW264.7 cells untreated or treated with DMXAA for 16h. Data represent the mean ± SEM. Statistical significance was analyzed by two-way ANOVA (n = 3), ns = not significant, ∗p < 0.05, ∗∗p < 0.01. (B) Protein levels of STAT1 in spleen lysates from 6-month-old WT and *C9orf72*^*−/−*^ mice were analyzed using western blot. Mixed male and female mice were used. Data represent the mean ± SEM. Statistical significance was analyzed by unpaired one-tail Student’s *t* test (n = 4), ns = not significant, ∗p < 0.05. (C) Protein levels of STAT1 and p-STAT1 in control or *C9orf72*^*−/−*^ RAW264.7 cells untreated or treated with DMXAA for 2h were analyzed using western blot and normalized to GAPDH. Data represent the mean ± SEM. Statistical significance was analyzed by unpaired two-tail Student’s *t* test (n = 3), ns = not significant, ∗p < 0.05. (D) RT-qPCR analysis of *Stat1* levels in control and *C9orf72*^*−/−*^ RAW264.7 cells untreated or treated with DMXAA for 16h. Data represent the mean ± SEM. Statistical significance was analyzed by two-way ANOVA (n = 3), ns = not significant, ∗p < 0.05, ∗∗∗∗p < 0.0001. (E) IFN-β levels in control and *C9orf72*^*−/−*^ RAW264.7 cells untreated or treated with DMXAA for 16h were measured using ELISA. Data represent the mean ± SEM. Statistical significance was analyzed by two-way ANOVA (n = 3), ns = not significant, ∗∗p < 0.01.

Since the degradation and trafficking of STING are not affected in *C9orf72*^*−/−*^ cells, next we examined whether STING transcription is altered by C9ORF72 deficiency. We found that STING transcription is significantly upregulated in *C9orf72*^*−/−*^ cells after DMXAA treatment (Figure 3A), indicating that C9ORF72 deficiency results in alterations in the pathway(s) upstream of STING instead of affecting STING signaling directly. Since STING transcription is partially regulated by the transcriptional factor STAT1,^27^ we next investigated alterations in STAT1 levels under C9ORF72 deficient conditions. A significant increase in the protein levels of STAT1 was found in lysates derived from spleens of *C9orf72*^*−/−*^ mice (Figure 3B) and *C9orf72*^*−/−*^ RAW264.7 cells (Figure 3C). In addition, a more dramatic increase in the levels of phosphorylated STAT1 (p-STAT1) was observed in *C9orf72*^*−/−*^ RAW264.7 cells after DMXAA treatment compared to the increase in STAT1 levels (Figure 3C), indicating that the JAK activities are hyper upregulated in the absence of C9ORF72 to mediate STAT1 phosphorylation. Moreover, RT-qPCR analysis revealed a significant increase in the mRNA levels of *Stat1* in *C9orf72* deficient RAW264.7 cells in response to DMXAA (Figure 3D), indicating that C9ORF72 might affect *Stat1* transcription in response to STING activation.

It is well known that the JAK-STAT pathway is activated by type-I IFNs^28^ and DMXAA is a type-I IFN inducer.^29^ Increased levels of STAT1 protein and mRNA in *C9orf72*^*−/−*^ cells with DMXAA treatment led us to hypothesize that type-I IFN levels might be altered in *C9orf72*^*−/−*^ cells. Indeed, we observed a significant increase in the levels of secreted IFN-β in *C9orf72*^*−/−*^ RAW264.7 cells after DMXAA treatment (Figure 3E), suggesting that C9ORF72 deficiency results in elevated type-I IFN production, which could lead to increased STAT1 levels and JAK/STAT signaling.

### Inhibition of JAK signaling rescues inflammatory phenotypes associated with C9ORF72 loss

**Figure 4:**
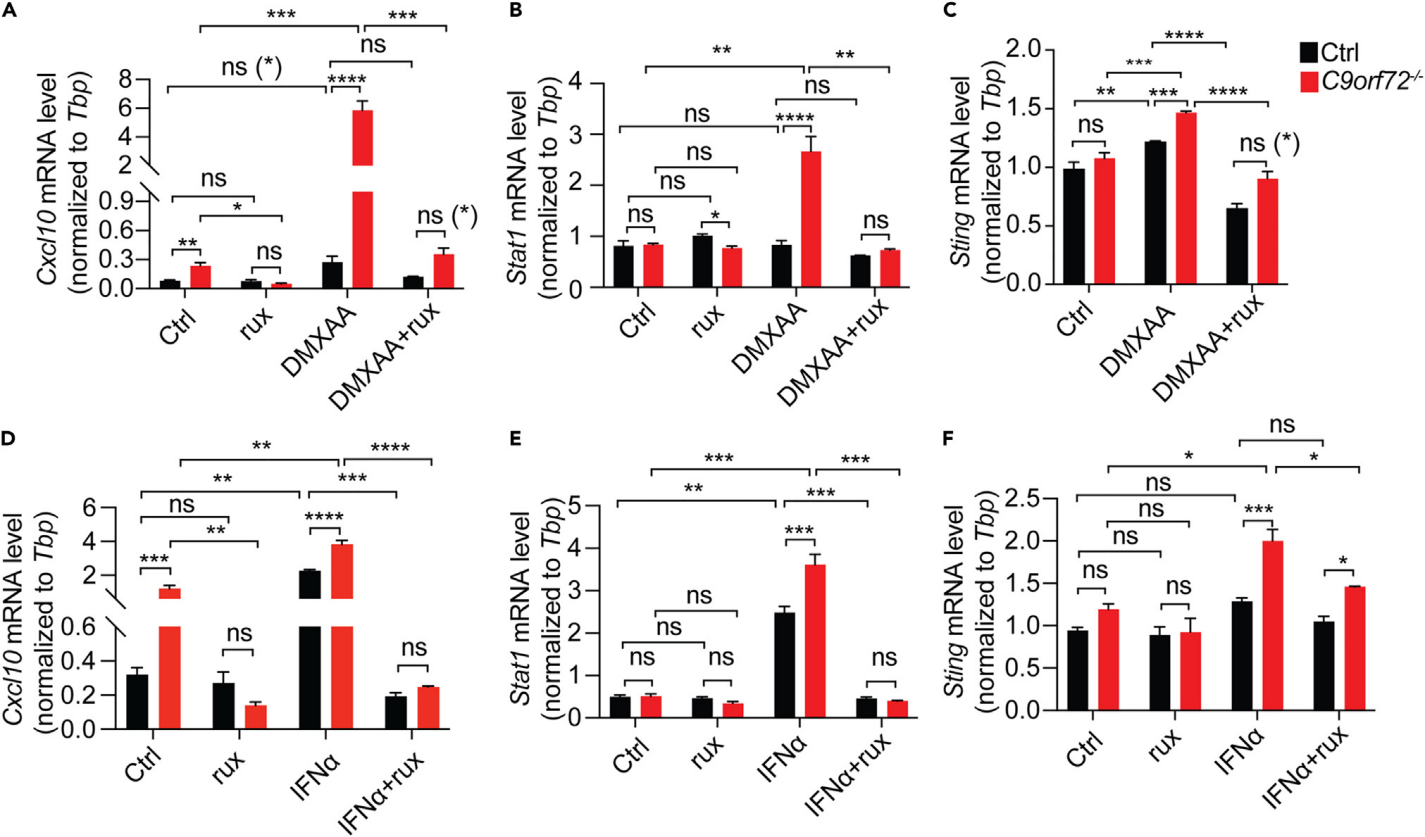
JAK-STAT inhibition partially rescues hyperactive inflammatory responses in *C9orf72*^*−/−*^ RAW264.7 cells (A-F) Control or *C9orf72*^*−/−*^ RAW264.7 cells were treated with or without ruxolitinib (1 μM) for 16hrs and then treated with DMXAA (A-C) or IFNα (1000 U/mL) (D-F) for additional 16hrs with or without ruxolitinib. *Cxcl10*, *Stat1,* and *Sting* mRNA levels were assayed by RT-qPCR. Data represent the mean ± SEM. Statistical significance was analyzed by two-way ANOVA (n = 3). Groups that exhibit non-significant differences with two-way ANOVA were re-analyzed using unpaired two-tailed Student’s *t* test, and the results are indicated in parentheses. ns = not significant, ∗p < 0.05, ∗∗p < 0.01, ∗∗∗p < 0.001, ∗∗∗∗p < 0.0001.

To confirm whether the increase in inflammatory responses under C9ORF72 deficient conditions is due to the activation of the JAK-STAT pathway, we treated C9ORF72 deficient cells with ruxolitinib, a widely used inhibitor of JAK1/2 kinase activities.^30^ Ruxolitinib treatment significantly rescued the increase in mRNA levels of *Cxcl10* (Figure 4A), *Stat1* (Figure 4B), and *Sting* (Figure 4C) in *C9orf72*^*−/−*^ cells after DMXAA treatment. These results support that the hyperactive JAK-STAT pathway causes elevated inflammatory responses under C9ORF72 deficient conditions. It should be noted that ruxolitinib treatment does not fully rescue the increases in mRNA levels of *Cxcl10* and *Sting* in *C9orf72*^*−/−*^ RAW264.7 cells in response to DMXAA (Figures 4A and 4C), consistent with the complicated regulatory system of STING transcription, in which other transcription factors besides STAT1, such as CREB or c-Myc are involved.^31^

In addition, we found that C9ORF72 deficiency results in exacerbated inflammatory responses to IFNα, a type-I IFN, as shown by elevated mRNA levels of *Stat1*, *Sting*, and *Cxcl10* (Figures 4D–4F). Inhibition of JAK activities with ruxolitinib fully rescues the increase in *Stat1* levels and partially rescues the increase in *Cxcl10* and *Sting* levels (Figures 4D–4F), indicating that the JAK-STAT signaling is elevated under C9ORF72 deficient condition in response to type-I IFN stimulation.

**Figure 5:**
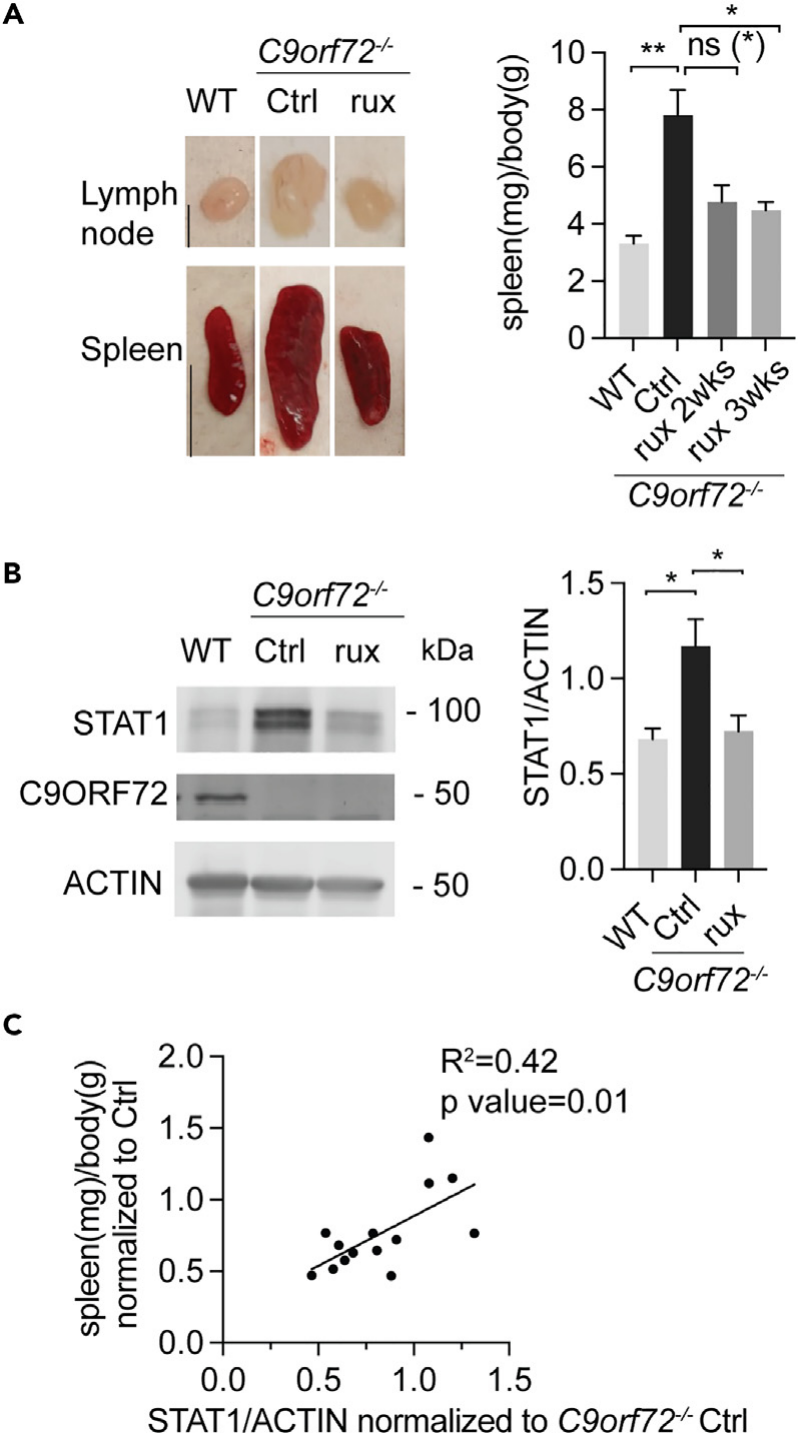
JAK1 inhibition rescues spleen and lymph node enlargement in *C9orf72*^*−/−*^ mice (A) 2.6–2.8 month-old WT mice were treated with DMSO (n = 4) and *C9orf72*^*−/−*^ mice were treated with either DMSO (n = 6), or with ruxolitinib (90 mg/kg daily) for 2 weeks (n = 3), or 3 weeks (n = 5). Representative image of lymph nodes (scale bar = 2 mm) and spleens (scale bar = 1 cm) from 2.6–2.8 month-old DMSO-treated WT mice or DMSO, or ruxolitinib (3 weeks) treated *C9orf72*^*−/−*^ mice are shown (left). The spleen weight (mg) over body weight (g) is quantified and shown on the right. Data represent the mean ± SEM. Statistical significance was analyzed by one-way ANOVA, n = 4–6. Groups that exhibit non-significant differences with two-way ANOVA were re-analyzed using unpaired two-tailed Student’s *t* test, and the results are indicated in parentheses. ns = not significant, ∗p < 0.05, ∗∗p < 0.01. (B) Western blot analysis of STAT1 protein levels in spleen samples for experiments in (A). Samples from *C9orf72*^*−/−*^ mice treated with ruxolitinib for 3 weeks were used. Data represent the mean ± SEM. Statistical significance was analyzed by one-way ANOVA, ns = not significant, n = 4–6, ∗p < 0.05. (C) Correlation graph between spleen weight and STAT1 protein levels in DMSO or ruxolitinib treated *C9orf72*^*−/−*^ mice. Pearson correlation coefficient (*r*) and significance (*p*) were calculated. n = 14.

We then investigated whether ruxolitinib can rescue the hyperactivated JAK-STAT pathway in response to C9ORF72 loss *in vivo*. *C9orf72*^*−/−*^ mice were treated with ruxolitinib for 2 weeks or 3 weeks and inflammatory phenotypes were analyzed. Remarkably, splenomegaly and lymphadenopathy in *C9orf72*^*−/−*^ mice were greatly reduced after 2 weeks of ruxolitinib treatment and significantly rescued after 3 weeks of ruxolitinib treatment (Figure 5A). The protein level of STAT1 in the spleen lysate of *C9orf72*^*−/−*^ mice also decreased significantly with ruxolitinib treatment compared to the DMSO control (Figure 5B). In addition, a significant correlation between spleen size and STAT1 level has been observed in *C9orf72*^*−/−*^ mice, despite a relatively low R^2^ between spleen size and STAT1 level due to variabilities (Figure 5C).

Taken together, our results suggest that C9ORF72 deficiency causes elevated JAK-STAT signaling, resulting in increased STAT1 levels and IFN production, which further elevates inflammatory signaling via a positive feedback loop under C9ORF72 deficient conditions.

### C9ORF72 deficiency causes impairment of lysosome integrity

**Figure 6:**
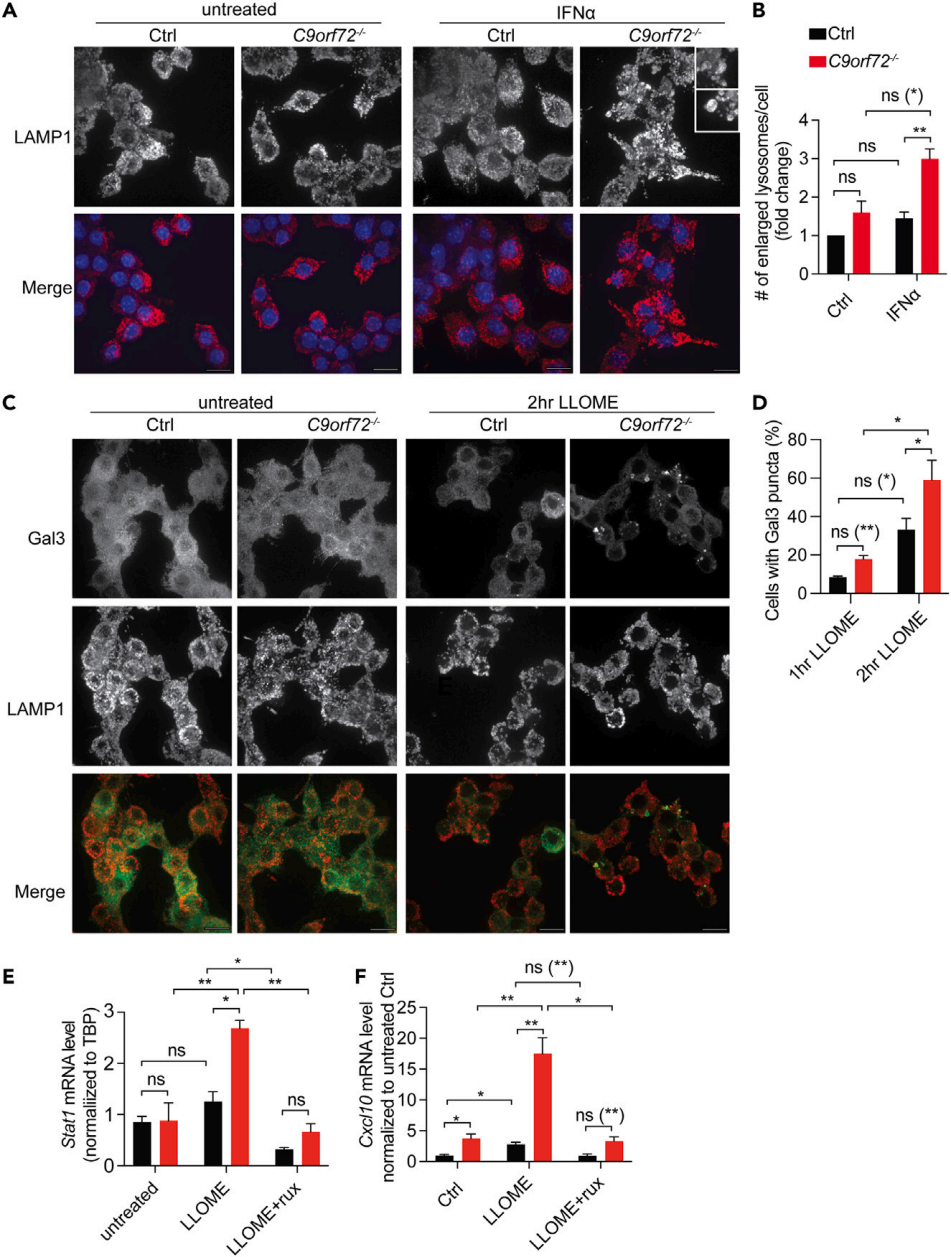
Impairment of lysosome integrity due to C9ORF72 loss results in increased JAK-STAT-mediated inflammation (A and B) Immunostaining of lysosome marker LAMP1 in control or *C9orf72*^*−/−*^ RAW264.7 cells without and with 16h of IFNα treatment (scale bar = 10 μm). The number of lysosomes with a diameter larger than 0.75 μm in each cell was counted and normalized to untreated control RAW264.7 cells. 106–142 cells from three independent experiments (142 (Ctrl); 178 (Ctrl IFNa); 106 (*C9orf72*^*−/−*^ Ctrl); 117 (*C9orf72*^*−/−*^ IFNα)) were quantified for the experiment in (A). Data represent the mean ± SEM. Statistical significance was analyzed by two-way ANOVA. Groups that exhibit non-significant differences with two-way ANOVA were re-analyzed using unpaired two-tailed Student’s *t* test, and the results are indicated in parentheses. ns = not significant, ∗∗p < 0.01. (C and D) Immunostaining of Galectin 3 and LAMP1 in control or *C9orf72*^*−/−*^ RAW264.7 cells without and with 2h LLOME (1 μM) treatment (scale bar = 10 μm). The number of cells with Galectin-3 puncta was quantified and normalized to the total number of cells in each condition. Total number of cells from four independent experiments analyzed: Ctrl 1h LLOME n = 525, *C9orf72*^*−/−*^ 1h LLOME n = 492, Ctrl 2h LLOME n = 319, *C9orf72*^*−/−*^ 2h LLOME n = 294. Data represent the mean ± SEM. Statistical significance was analyzed by two-way ANOVA. Groups that exhibit non-significant differences with two-way ANOVA were re-analyzed using unpaired two-tailed Student’s *t* test, and the results are indicated in parentheses. ns = not significant, ∗p < 0.05. (E and F) Control or *C9orf72*^*−/−*^ RAW264.7 cells are either untreated (Ctrl), or treated with 4hrs of LLOME then recover for 12hrs in normal cell medium (LLOME), or treated with ruxolitinib for 16hrs before 4 h of LLOME treatment and then recover for 12hrs in normal cell medium with ruxolitinib (LLOME+rux). The mRNA level of *Stat1* and *Cxcl10* was analyzed by RT-qPCR. Data represent the mean ± SEM. Statistical significance was analyzed by two-way ANOVA (n = 3). Groups that exhibit non-significant differences with two-way ANOVA were re-analyzed using unpaired two-tailed Student’s *t* test, and the results are indicated in parentheses. ns = not significant, ∗p < 0.05, ∗∗p < 0.01.

Lysosome dysregulation was found to be one of the causes of inflammation. Undegraded substrates such as DNA or lysosome hydrolases leaked out from damaged lysosomes have been shown to induce inflammatory responses.^32^^,^^33^ Previous studies have reported lysosome deficits under C9ORF72 deficient conditions.^10^^,^^13^^,^^18^^,^^34^ We observed an increased number of enlarged lysosomes in *C9orf72*^*−/−*^ RAW264.7 cells after IFNα treatment (Figures 6A and 6B). Enlarged lysosomes could be a consequence of accumulated undigested materials,^35^ which can induce cell stress and/or inflammation if released into the cytosol. Moreover, we found that lysosomes in *C9orf72*^*−/−*^ RAW264.7 cells are more sensitive to lysosome permeabilization compared to control cells. In response to treatment with L-Leucyl-L-Leucine methyl ester (LLOME), a lysosome damaging reagent. *C9orf72*^*−/−*^ RAW264.7 cells showed an increased number of cells with lysosomes labeled by Galectin 3 (Gal3): a lectin recruited to lysosomes due to the exposure of lysosomal glycoproteins caused by lysosomal membrane permeabilization (Figures 6C and 6D).

To confirm whether lysosome leakage could lead to inflammation in *C9orf72*^*−/−*^ cells, RT-qPCR was used to examine changes in the mRNA levels of *Stat1* and *Cxcl10* in control or *C9orf72*^*−/−*^ RAW264.7 cells with or without LLOME treatment. LLOME treatment leads to a significant elevation of *Stat1* (Figure 6E) and *Cxcl10* (Figure 6F) mRNA levels in *C9orf72*^*−/−*^ cells. This increase is rescued by ruxolitinib treatment, indicating that lysosome damage could cause JAK-dependent inflammatory response under C9ORF72 deficient conditions.

## Discussion

**Figure 7:**
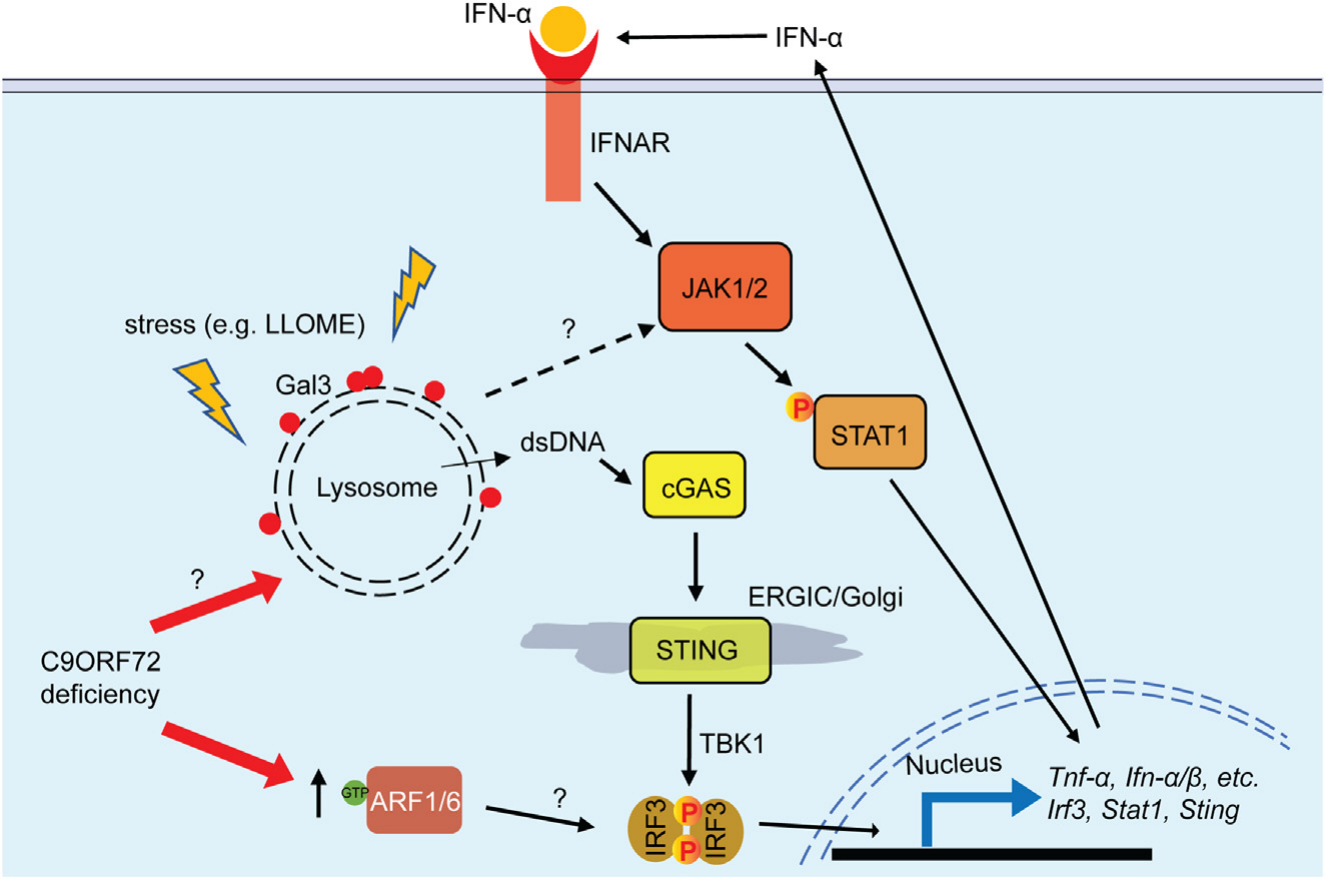
Graphical summary of the positive feedback loop that leads to the excessive inflammatory response under C9ORF72 deficient conditions Lysosomes are prone to permeabilization under stress under C9ORF72 deficient conditions, although the exact mechanism is still unknown. Undegraded materials, lysosomal hydrolases, or ROS released from permeabilized lysosomes could activate the JAK-STAT pathway and leaked dsDNAs could activate the cGAS/STING pathway, leading to elevated inflammation and increased expression of cytokines. The increase in cytokine levels further activates the JAK-STAT pathway, stresses the lysosome, and exacerbates lysosome permeabilization in C9ORF72 deficient cells through unknown mechanisms. Furthermore, alteration in the activities of the ARF1/6 GTPase activities under C9ORF72 deficient conditions may lead to increased phosphorylation and activation of IRF3, a transcription factor involved in the expression of multiple cytokines, resulting in a further increase in inflammatory responses.

In this study, we found an interesting link between C9ORF72, lysosomal dysfunction, and inflammatory signaling (Figure 7): (1) C9ORF72 deficiency results in lysosomal dysfunction and impairment of lysosomal membrane integrity (Figure 6); (2) Lysosomal leakage in C9ORF72 deficient cells in response to lysosomal perturbations triggers inflammatory responses in a JAK/STAT dependent manner (Figures 6C–6F), resulting in increased levels of inflammatory cytokines; (3) Levels of inflammatory mediators, IRF3, STING, and STAT1 are significantly increased under C9ORF72 deficient conditions (Figures 1C, 1D, 2A, 2B, 3B, and 3C); (4) Inhibition of JAK/STAT signaling with the JAK inhibitor ruxolitinib rescues inflammatory phenotypes and increased STAT1 levels in C9ORF72 deficient cells and mice (Figures 4 and 5).

### C9ORF72 deficiency results in the hyperactivation of the JAK-STAT pathway

While exploring the inflammatory pathways regulated by C9ORF72, we demonstrated that C9ORF72 deficiency leads to the hyperactivation of the JAK-STAT pathway, in addition to the endosomal TLR signaling pathways and the cGAS-STING signaling pathway previously reported.^17^^,^^18^ A significant increase in STAT1 levels was observed in C9ORF72 deficient cells and tissues (Figure 3), which may result in enhanced signal transduction involving STAT1. For example, activation of the JAK-STAT pathway via IFNα treatment results in enhanced mRNA levels of *Stat1*, *Sting*, and *Cxcl10* in *C9orf72*^*−/−*^ cells, which can be fully or partially rescued by inhibiting JAK1/2 kinase activities (Figures 4D–4F).

In addition, *Cxcl10* levels are increased in *C9orf72*^*−/−*^ cells without any treatment, and this increase is rescued by ruxolitinib treatment, indicating that the JAK-STAT pathway is activated in *C9orf72*^*−/−*^ cells without any stimuli (Figures 4A and 4D). More importantly, ruxolitinib treatment rescues splenomegaly and lymphadenopathy observed in *C9orf72*^*−/−*^ mice (Figure 5), confirming the importance of the JAK-STAT activation in the inflammatory responses under C9ORF72 deficient conditions. It should be noted that ruxolitinib inhibits both JAK1 and JAK2 and also alleviates inflammatory responses in patients with *STAT1* or *STAT3* gain-of-function variants.^36^ Thus, the strong anti-inflammatory effect of ruxolitinib that we observed under the C9ORF72 deficient condition may not be solely due to its inhibition of JAK-STAT1 and other JAK-STAT pathways might be affected by C9ORF72 deficiency. Nevertheless, JAK and/or STAT dysregulations are found to cause multiple autoimmune diseases such as rheumatoid arthritis, and inhibitors of the JAK-STAT pathway are used to treat these diseases.^37^ Interestingly, a newly developed machine learning framework, DRIAD (Drug Repurposing In Alzheimer’s Disease), identified multiple drugs that inhibit one or more proteins in the JAK family as top-scoring drugs for treating Alzheimer’s disease.^38^ In addition, ALS/FTLD patients with *C9ORF72* HRE expansion were found to harbor cytoplasmic double-stranded RNA (dsRNA), an established trigger of innate immunity, and inhibition of JAK/STAT signaling by ruxolitinib was shown to reverse cell death triggered by dsRNA in cultured human neurons.^39^ Based on these studies, baricitinib, an FDA-approved JAK inhibitor, is currently in the clinical trial for people with mild cognitive impairment, ALS, or ALS patients carrying *C9ORF72* mutant (NCT05189106). Our demonstration of JAK-STAT hyperactivation under C9ORF72 deficient conditions may further explain the relatively high occurrence of autoimmune diseases in ALS/FTLD patients with *C9ORF72* mutations^16^ and support the inhibition of the JAK/STAT pathway as a valid therapeutic approach to treat these patients.

### Regulation of STING levels and signaling by C9ORF72

Consistent with a previous report, we have found an increase in STING protein levels under C9ORF72 deficient conditions.^17^ However, in contrast to the previous report, we did not observe a defect in STING degradation in C9ORF72 deficient cells (Figure 2C). We explored whether the increase in the levels of STING proteins could be due to transcriptional upregulation. However, *Sting* mRNA levels do not seem to be significantly altered in C9ORF72 deficient cells under normal conditions, although there is a trend of increase under some conditions (Figures 3A, 4C, and 4F). In contrast, *Sting* mRNA levels are significantly increased in *C9orf72*^*−/−*^ cells in response to DMXAA treatment (Figures 3A and 4C), consistent with a profound increase in p-STAT1 levels in these cells with DMXAA (Figure 3C). Interestingly, inhibition of JAK signaling with ruxolitinib treatment only partially suppresses this increase in *Sting* transcription (Figures 4C and 4F), indicating that there might be other factors, such as CREB or c-Myc involved in regulating *Sting* mRNA levels.^31^ In addition, since both *Sting* mRNA levels (Figures 3A, 4C, and 4F) and STING protein degradation (Figure 2C) are not significantly altered by C9ORF72 deficiency under normal conditions, the mechanisms leading to increased STING protein levels in C9ORF72 deficient cells remain to be determined.

### Regulation of IRF3 levels by C9ORF72

Our study also showed a significant increase in the mRNA and protein levels of the transcription factor IRF3 in C9ORF72 deficient Raw264.7 cells (Figures 1C and 1D). Interestingly, a recent study identified several small GTPases, including ARF1 and ARF6, that can stimulate IRF3 phosphorylation.^40^ C9ORF72 is known to form a complex with two other cytosolic proteins, Smith-Magenis Chromosome Regions 8 (SMCR8) and WD repeat-containing protein (WDR41)^10^^,^^23^^,^^34^^,^^41^ to function as a GTPase accelerating protein (GAP) for ARF1 and ARF6 GTPases.^21^ Thus, the increased levels of *p*-IRF3 in *C9orf72*^*−/−*^ cells (Figure 1D) could be due to the enhanced levels of active Arf1 and/or Arf6 GTPases caused by loss of C9ORF72. IRF3 is a also cofactor for the induction of interferon-stimulated genes (ISGs).^42^ Upon phosphorylation and nuclear translocation, IRF3 can formenhanceosome with other transcription factors to induce IFN-β transcription.^43^ It should be noted that several other IRF family members, including IRF7 and 9, have also been shown to get upregulated under C9ORF72 deficient conditions and in ALS patients with *C9ORF72* HRE.^17^^,^^44^ IRF7 activation and dimerization can also induce IFN-α transcription.^43^

In addition, type-I IFN treatment leads to the upregulation of STAT1 levels (Figure 4E). STAT1 is known to upregulate its own transcription once activated.^28^ Furthermore, STAT1 together with STAT2 and IRF9 forms Interferon-stimulated gene factor 3 (ISGF3), leading to the transcription of IRF7 and other ISGs.^45^ STING and IRF3 have also been identified as ISGs, thus their expression may also be elevated by interferons.^27^^,^^46^ Additionally, there are many cross-talks between different immune signaling pathways. For example, IRF3 also functions downstream of TLR signaling and cytosolic DNA/RNA sensing utilizing STING and other receptors.^47^ It is conceivable that the increase in IRF levels together with the increase in STAT1 levels greatly sensitizes the C9ORF72 deficient cells to inflammatory responses to many different stimuli.

### Impaired lysosomal integrity in C9ORF72 deficient cells

C9ORF72 deficiency has long been associated with various lysosomal defects including lysosome enlargement, poor lysosome acidification, and a decrease in autophagosome-lysosome fusion, and so forth.^10^^,^^13^^,^^18^^,^^34^ Here, we show that loss of C9ORF72 results in impaired lysosome membrane integrity under stress conditions. Since the lysosome is the degradation center of the cell, maintaining lysosomal membrane integrity is vital to prevent leakage of undegraded materials and lysosomal hydrolases that are toxic to the cells. The increased lysosome membrane permeabilization after LLOME treatment in C9ORF72 deficient cells suggests that C9ORF72 is critical for the maintenance of lysosomal membrane integrity. Although the exact mechanism is still unknown, it may be related to the function of the C9ORF72/SMCR8/WDR41 complex as a GAP and/or GEF of small GTPases ARF and/or RAB.^48^ The C9ORF72 complex is recruited to the lysosome under amino acid-deprived conditions by interacting with PQLC2, a transporter on the lysosomal membrane.^49^ This recruitment may act as a switch to switch on or off activities of C9ORF72 toward one or more small GTPase. For example, the C9ORF72 complex has been shown to function as a GAP for ARF1 GTPase,^21^- thus by recruiting the complex to the lysosome, its GAP activity toward ARF1 is inhibited because ARF1 is localized to the Golgi. On the other hand, the lysosomelocalized C9ORF72 complex might affect the activities of RAB7 GTPase, which localizes to the lysosome^50^ and interacts with C9ORF72^51^^,^^52^. Future work is needed to identify the GTPase(s) and mechanisms regulated by the C9ORF72 complex to affect lysosomal membrane integrity.

Another question that remains to be answered is the exact mechanism of how lysosome permeabilization leads to the activation of the JAK/STAT pathway under the C9ORF72 deficient condition. In this regard, it has been shown that lysosome substrate overload and/or lysosome permeabilization can lead to the generation of reactive oxygen species (ROS)^53^^,^^54^ and elevated levels of ROS can lead to the activation of the JAK/STAT pathway.^55^ For example, STAT3 can be activated by oxidative stress caused by the deficiency of AEP, a lysosomal asparagine endopeptidase, and STAT3 activation can in turn promote lysosomal hydrolase expression.^53^ Interestingly, C9ORF72 deficiency has been shown to increase ROS levels in the iPSC-derived motor neurons from multiple ALS/FTLD patients with *C9ORF72* mutations^56^ and in *C9orf72*^*−/−*^ bone marrow-derived primary macrophages after zymosan ingestion.^11^ Thus, the elevated ROS levels due to lysosome leakage could lead to the activation of the JAK-STAT pathway under the C9ORF72 deficient condition.

### Limitations of the study

While our studies show a critical role of C9ORF72 in suppressing JAK/STAT mediated inflammation, the exact mechanism of how C9ORF72 deficiency leads to JAK-STAT1 activation awaits further investigation. One possibility is that JAK/STAT is activated by ROS and undegraded substrates from permeabilized lysosomes in *C9orf72*^*−/−*^ cells since we as well as others have observed the lysosome defects and decreased lysosomal membrane integrity in *C9orf72*^*−/−*^ cells. Nevertheless, the precise mechanisms connecting lysosome defects and JAK-STAT1 activation, as well as the function of the C9ORF72 complex in maintaining proper lysosomal function and lysosomal membrane integrity, are still elusive. Another potential avenue through which C9ORF72 may affect inflammation is via IRF3, as we have observed increased levels of IRF3 and phospho-IRF3 under C9ORF72 deficient conditions. However, the mechanisms by which C9ORF72 regulates IRF3 levels remain to be investigated. Considering that ARF1 and ARF6 have been shown to stimulate IRF3 phosphorylation^40^ and the C9ORF72 complex functions as a GAP for ARF1 and ARF6 GTPases,^21^ we propose that C9ORF72 might regulate IRF3 signaling via ARF1 and ARF6. However, the mechanism by which how ARF1/6 GTPases regulate IRF3 phosphorylation, and whether ARF1/6 GTPases are responsible for increased IRF3 levels in *C9orf72*^*−/−*^ cells, need to be determined. Moreover, the exact cause of the increase in STING protein level under C9ORF72 deficient conditions remains unclear. Addressing these missing links will provide further insights into the molecular and cellular mechanisms by which C9ORF72 regulates inflammation.

## STAR★Methods

### Key resources table

**Table:** 

REAGENT or RESOURCE	SOURCE	IDENTIFIER
**Antibodies**		

rabbit anti-STING	Proteintech	Cat#19851-1-AP; RRID:AB_10665370
rabbit anti- C9ORF72	Proteintech	Cat# 22637-1-AP, RRID: AB_10953528
mouse anti-ACTIN	Proteintech	Cat# 66009-1-Ig, RRID: AB_2687938
mouse anti-GAPDH	Proteintech	Cat# 60004-1-Ig, RRID: AB_2107436
rabbit anti-STAT1	Proteintech	Cat# 10144-2-AP, RRID: AB_2286875
mouse anti-C9ORF72	GeneTex	Cat# GTX632041, RRID: AB_2784546
rabbit anti-IRF3	Cell Signaling Technology	Cat# 4302, RRID: AB_1904036
rabbit anti-p-IRF3	Cell Signaling Technology	Cat# 4947, RRID: AB_823547
rabbit anti-pSTAT1	R and D Systems	Cat# AF2894, RRID: AB_2198137
mouse anti-ACBD3	Santa Cruz Biotechnology	Cat# sc-101277, RRID: AB_2273355
mouse anti-Galectin3	BioLegend	Cat# 126701, RRID: AB_1134255
rat anti-mouse LAMP1	BD Biosciences	Cat# 553792, RRID: AB_2134499
IRDye 800CW Donkey anti-Mouse IgG	LI-COR Biosciences	Cat# 926-32212, RRID: AB_621847
IRDye 680RD Donkey anti-Rabbit IgG	LI-COR Biosciences	Cat# 926-68073, RRID: AB_10954442
Donkey anti-Goat IgG Alexa Fluor 680	Thermo Fisher Scientific	Cat# A-21084, RRID: AB_2535741
Donkey anti-Rabbit IgG Alexa Fluor 680	Thermo Fisher Scientific	Cat# A10043, RRID: AB_2534018
Donkey anti-Mouse IgG Alexa Fluor 680	Thermo Fisher Scientific	Cat# A10038, RRID: AB_2534014
Donkey anti-Mouse IgG Alexa Fluor 568	Thermo Fisher Scientific	Cat# A10037, RRID: AB_2534013
Donkey anti-Rabbit IgG Alexa Fluor 488	Thermo Fisher Scientific	Cat# A32790TR, RRID: AB_2866495
Donkey anti-Mouse IgG Alexa Fluor 647	Thermo Fisher Scientific	Cat# A32787, RRID: AB_2762830
Donkey anti-Sheep IgG Alexa Fluor 647	Thermo Fisher Scientific	Cat# A-21448, RRID: AB_2535865
Donkey anti-Rat IgG Alexa Fluor 680	Jackson ImmunoResearch Labs	Cat# 712-625-153, RRID: AB_2340699

**Chemicals, peptides, and recombinant proteins**		

ruxolitinib	Advanced ChemBlocks	Cat# G-6185
ruxolitinib	Cayman Chemical	Cat# 11609
LLOME	Cayman Chemical	Cat# 16008
DMXAA	Cayman Chemical	Cat# 14617
IFNα	BioLegend	Cat# 752802
Poly (I:C)	Invivogen	Cat# tlrl-pic
LPS	Sigma-Aldrich	Cat# L2630
Imiquimod	Cayman Chemical	Cat# 14956
ODN 2395	Invivogen	Cat# tlrl-2395

**Critical commercial assays**		

ELISA MAX™ Standard Set Mouse TNF-α	BioLegend	Cat#430901
Mouse IFN-beta DuoSet ELISA	R and D Systems	Cat#DY8234-05
SuperScript™ III Reverse Transcriptase	Thermo Fisher Scientific	Cat#18080044
Bradford protein assay	Bio-Rad	Cat# 5000002

**Experimental models: Cell lines**		

RAW264.7	ATCC	Cat# TIB-71, RRID:CVCL_0493
HEK293T	ATCC	RRID:CVCL_0063

**Experimental models: Organisms/strains**		

Mouse: C57BL/6	Jackson Laboratory	RRID:IMSR_JAX:000664
Mouse: *C9orf72*^*-/-*^ C57BL/6	This paper	N/A

**Oligonucleotides**		

Mouse Genotyping Forward: ACCTGGAATGCAGTGAGACC	This paper	N/A
Mouse Genotyping Reverse: TGCCCAGGAGACACAACATA	This paper	N/A
mC9orf72 CRISPR guide RNA forward: caccgGTATCATTCCCATGCTTACT	This paper	N/A
mC9orf72 CRISPR guide RNA reverse: aaacAGTAAGCATGGGAATGATACc	This paper	N/A
mSTING Forward: AAATAACTGCCGCCTCATTG	This paper	N/A
mSTING Reverse: TGGGAGAGGCTGATCCATAC	This paper	N/A
mSTAT1 Forward: CTGAATATTTCCCTCCTGGG	This paper	N/A
mSTAT1 Reverse: TCCCGTACAGATGTCCATGAT	This paper	N/A
mIRF3 Forward: GGCTTGTGATGGTCAAGGTT	This paper	N/A
mIRF3 Reverse: CATGTCCTCCACCAAGTCCT	This paper	N/A
mCXCL10 Forward: GTGCTGCCGTCATTTTCT	This paper	N/A
mCXCL10 Reverse: CCCTATGGCCCTCATTCT	This paper	N/A
mIL1-β Forward: GCAACTGTTCCTGAACTCAACT	This paper	N/A
mIL1-β Reverse: ATCTTTTGGGGTCCGTCAACT	This paper	N/A
mTBP Forward: CCCCACAACTCTTCCATTCT	This paper	N/A
mTBP Reverse: GCAGGAGTGATAGGGGTCAT	This paper	N/A
Actin Forward: ACGAGGCCCAGAGCAAGAG	This paper	N/A
Actin Reverse: TCTCCAAGTCGTCCCAGTTG	This paper	N/A

**Recombinant DNA**		

Plasmid: pMD2.G	pMD2.G was a gift from Didier Trono	Cat# 12259 RRID: Addgene_12259
Plasmid: psPAX2	psPAX2 was a gift from Didier Trono	Cat# 12260 RRID: Addgene_12260

**Software and algorithms**		

GraphPad Prism 9	Graphpad Software	https://www.graphpad.com/
ImageJ	NIH	RRID:SCR_003070
Image Studio Lite	LI-COR Biosciences	RRID:SCR_013715
SlideBook		RRID:SCR_014300

### Resource availability

#### Lead contact

Further information and requests for resources and reagents should be directed to and will be fulfilled by the lead contact, Fenghua Hu (fh87@cornell.edu).

#### Materials availability

Plasmids and cell lines generated in this study are available from the lead contact upon request.

### Experimental model and subject details

#### Mouse strains

*C9orf72* knockout mice were generated using CRISPR/Cas9 genome editing with a guide RNA (gRNA) targeting exon 2 of mouse gene 3110043O21RIK as previously described,^10^ Offspring from the founder containing 108 bp deletion (including the start codon) were backcrossed to C57BL/6 for another five generations and used in the study. Mixed female and male mice were used. The age of the mice used in each experiment is indicated in the figure legend. The animal protocol (2017-0056) was approved by Cornell University’s animal care and use committee following the National Research Council’s guide to the care of laboratory animals.

#### Cell culture

HEK293T and RAW264.7 cells were maintained in Dulbecco’s Modified Eagle’s Medium (Cellgro) supplemented with 10% fetal bovine serum (Gibco) and 1% Penicillin–Streptomycin (Invitrogen) in a humidified incubator at 37 °C and 5% CO_2_. RAW264.7 cells with C9ORF72 deletion or controls were generated by infecting the cells with lentivirus expressing Cas9 and gRNAs (GTATCATTCCCATGCTTACT) targeted to mouse *C9orf72* exon 4. Cells were selected with puromycin (2ug/mL) two days after infection and the knockout was confirmed by western blot analysis. The cells were tested negative for mycoplasma contamination.

### Method details

#### Mouse treatment

Ruxolitinib was first dissolved in DMSO (1mg/uL) and then further diluted in 0.5% methylcellulose saline by sonication before use. DMSO diluted in 0.5% methylcellulose saline was used as control. Two-month-old mice (weighing around 20 g at the time of treatments) were treated with DMSO or 90 mg/kg ruxolitinib once daily by oral gavage for two or three weeks.

#### Protein analysis

Cells and tissues were lysed in RIPA buffer (50 mM Tris pH 8.0, 150 mM NaCl, 1% Triton X-100, 0.1% SDS and 0.1% deoxycholic acid) with protease and phosphatase inhibitors. Samples were denatured in 2xSDS sample buffer (4% SDS, 20% glycerol, 100 mM Tris pH 6.8, 0.2 g/L bromophenol blue) by boiling for 5 min. Samples were run on 12% polyacrylamide gels and transferred to PVDF membranes (Millipore). Membranes were blocked in either Odyssey Blocking Buffer (LI-COR Biosciences) or 5% non-fat milk in PBS for 1 h followed by incubation with primary antibodies overnight at 4°C. Membranes were washed 3 times with Tris-buffered saline with 0.1% Tween-20 (TBST) and then incubated with secondary antibodies for 2 h at room temperature. Membranes were washed 3 times with TBST and imaged using an Odyssey Infrared Imaging System (LI-COR Biosciences) with settings in the linear range of band intensities. Western blot signals were then analyzed using the Image J software.

#### Immunofluorescence microscopy

Cells grown on glass coverslips were fixed in 3.7% paraformaldehyde for 15 min, washed 3 times with PBS, and permeabilized and blocked in Odyssey Blocking Buffer with 0.05% saponin for 15 min. Primary antibodies diluted in blocking buffer with 0.05% saponin were applied to the cells overnight at 4°C. Coverslips were washed 3 times with PBS. Secondary antibodies and Hoechst stain diluted in blocking buffer with 0.05% saponin were applied to the cells for 2 h at room temperature. Coverslips were washed and mounted onto slides with Fluoromount G (Southern Biotech). Images were acquired on a CSU-X spinning disc confocal microscope (Intelligent Imaging Innovations) with an HQ2 CCD camera (Photometrics) using a 100x objective.

#### ELISA

Cell medium samples were collected and analyzed using ELISA Max Standard Set Mouse TNF-α kit from Biolegend and Mouse IFN-beta DuoSet ELISA from R&D Systems according to the manufacturer’s instructions.

#### RT-PCR

RNA was purified from RAW264.7 cells using TRIzol Reagent (Invitrogen). One microgram of total RNA was reverse transcribed using a poly(T) primer and SuperScript III Reverse Transcriptase (Invitrogen). qPCR was performed on a LightCycler 480 (Roche Applied Science), and transcript levels were calculated using efficiency-adjusted ΔΔ-CT. All transcripts were normalized to *Tbp*.

### Quantification and statistical analysis

#### Image analysis

For the quantitative analysis of enlarged lysosomes, the number of lysosomes with a diameter larger than 0.75 μm was counted manually. At least 50 cells were analyzed in each treatment per experiment and the experiment was independently repeated four times. For the quantitative analysis of cells with Galectin 3 puncta, the total number of cells and the number of cells with Galectin3 puncta were counted manually. Around 100 cells were counted in each treatment per experiment and the experiment was independently repeated three times.

#### Statistical analysis

The data were presented as mean ± SEM. Two-group analysis was performed using the Student’s *t* test. Two-way ANOVA followed by Bonferroni’s multiple comparison tests was used for multiple-group comparison. All statistical analyses were performed using GraphPad Prism version 9 software (GraphPad Software, San Diego, CA). p-values <0.05 were considered statistically significant.

## Data Availability

• Data reported in this paper and any additional information required to reanalyze the data reported in this paper are available from the lead contact upon request. • This paper does not report any original code.
